# A Complex Case of Laryngospasm Secondary to COVID-19 Infection in a 40-Year-Old Male

**DOI:** 10.7759/cureus.68828

**Published:** 2024-09-06

**Authors:** Asia M Eter, Tomohiro Yamamoto, Satoshi Yamamoto

**Affiliations:** 1 Anesthesiology, University of Texas Medical Branch at Galveston, Galveston, USA; 2 College of Medicine, University of Gunma, Maebashi, JPN

**Keywords:** covid-19, laryngospasm, pandemic, pleural effusion, pneumothorax, post-anesthesia, surgical patient

## Abstract

COVID-19 is a novel viral infection with a wide variety of clinical manifestations ranging from asymptomatic cases to severe respiratory illness. Laryngospasm is a spontaneous sustained closure of the laryngeal muscles leading to acute airway obstruction. We report a case of a 40-year-old male with a history of nephrolithiasis who underwent laparoscopic pyeloplasty and developed laryngospasm as a consequence of contracting COVID-19. The case was further complicated by the development of pneumothorax and pleural effusion during the postoperative period. The patient was managed with supplemental oxygen, antibiotics, antiviral therapy, and close monitoring. He recovered without any additional complications. This case highlights the potential initial clinical manifestation and the importance of early diagnosis and treatment of COVID-19 infection in surgical patients.

## Introduction

Laryngospasm is a rare complication that may be related to the induction and emergence phases of anesthesia [1]. The term laryngospasm refers to a reflexive, sustained closure of the laryngeal muscles that can be initiated by various types of irritation. The occurrence of laryngospasm has the potential to lead to acute desaturation and subsequent organ dysfunction resulting from the acute closure of the airway [2]. The emergence phase of anesthesia, when extubation takes place, involves coughing of secretions and is considered an aerosol-generating procedure [3]. This situation becomes particularly complex when dealing with a patient under infection control precautions, which may lead to a higher likelihood of reintubation.

The recent COVID-19 pandemic has presented novel challenges in the realm of anesthesia management, primarily due to the heightened susceptibility to infection and postoperative complications stemming from the immunosuppressive effects induced by surgery. Surgical stress can lead to elevated cortisol levels, which contribute to immunosuppression by decreasing lymphocyte proliferation and function [4]. COVID-19, an emerging viral disease, exhibits a diverse array of clinical presentations, ranging from asymptomatic instances to severe respiratory conditions. The accuracy of COVID-19 testing hinges significantly on prior exposure history and local disease prevalence, thereby impacting the interpretation of test results [5]. Given the highly transmissible nature of the virus [6], extubation for patients afflicted with COVID-19 is managed within airborne isolation facilities to mitigate the risk of aerosolization. Emphasis is placed on employing strategies for "smooth extubation" to reduce instances of coughing and agitation, thus minimizing the likelihood of laryngospasm development [7]. Herein, we present a clinical case involving a 40-year-old male who experienced laryngospasm, pneumothorax, pleural effusion, and subsequent COVID-19 infection after undergoing laparoscopic pyeloplasty, alongside an examination of potential etiological factors and management approaches for these complications.

## Case presentation

A 40-year-old male with a history of nephrolithiasis presented with recurrent left flank pain and hematuria. His medical history was notable for tobacco use, gastroesophageal reflux disease, two previous episodes of left ureteral stone passage, and a family history of nephrolithiasis. He had no history of alcohol use or chronic medical conditions and was not on any medications. Physical examination revealed a healthy individual with no acute distress. Vital signs were within normal limits, and abdominal and genitourinary examinations were unremarkable. Laboratory tests were normal, including complete blood count, serum chemistry, urinalysis, and coagulation profile. An initial preoperative COVID-19 test was negative. Imaging showed a left ureteropelvic junction (UPJ) obstruction with moderate hydronephrosis and a 5 mm stone in the lower pole of the left kidney. He was diagnosed with left UPJ obstruction and nephrolithiasis and subsequently scheduled for a laparoscopic pyeloplasty and stone removal.

**Figure 1:**
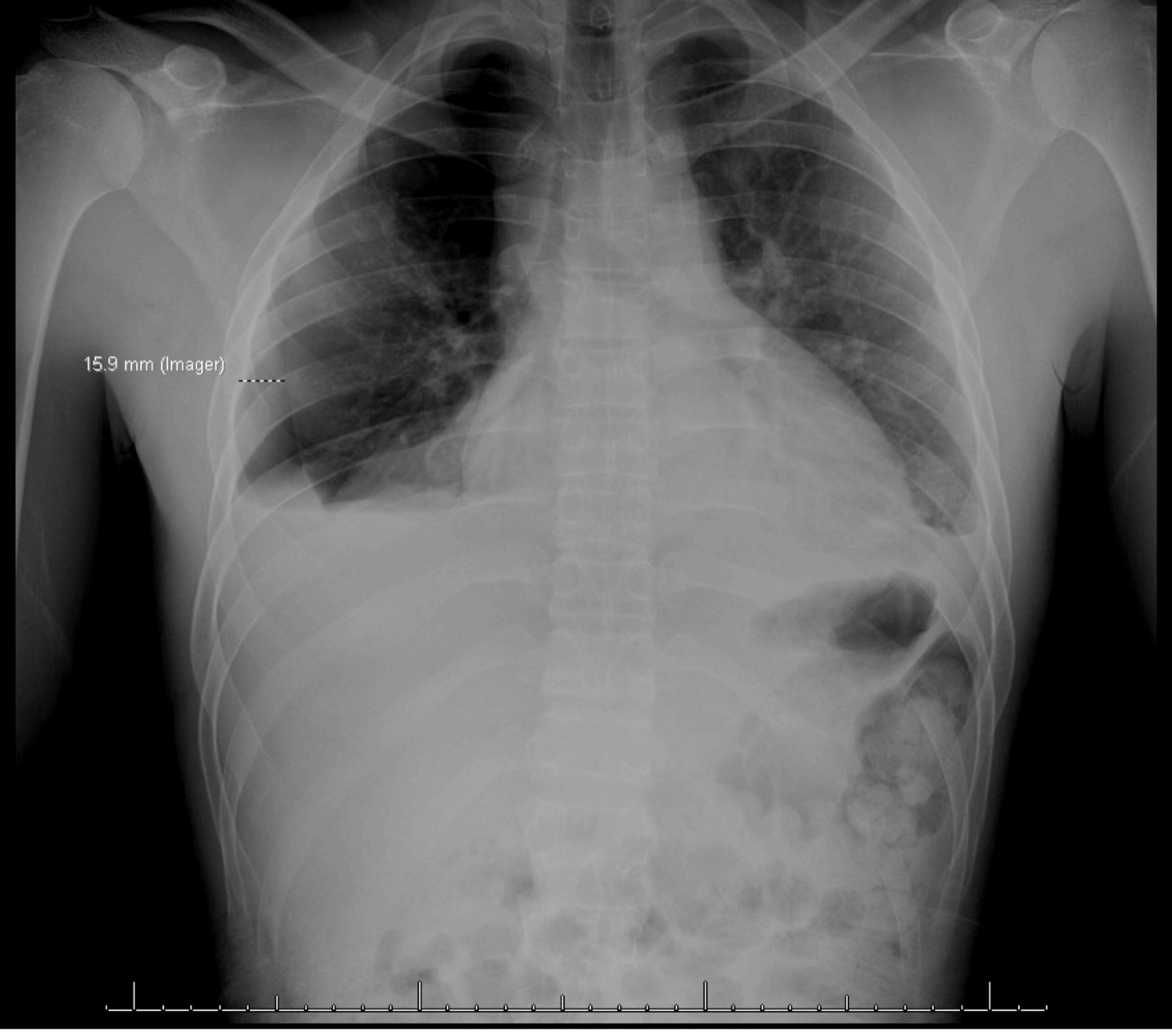
Chest X-ray showing the development of right pleural effusion overlying a pneumothorax, with a trace left pleural effusion also present.

The preoperative evaluation of the patients was uneventful, showing an American Society of Anesthesiologists (ASA) score of 2, Mallampati score of 2, thyromental distance exceeding 5 cm, intact neck mobility, and absence of significant dental issues. General anesthesia was induced with propofol, fentanyl, and rocuronium (0.6 mg/kg). The patient underwent endotracheal intubation through intravenous induction, using direct laryngoscopy with a Mac 3 blade (IntuBrite, Salter Labs, Arvin, California, United States). Maintenance was conducted utilizing sevoflurane in conjunction with volume-controlled ventilation. Following the conclusion of the surgical procedure, the Train of Four (TOF) ratio was determined to be 3/4, which later transitioned to 4/4 post-reversal facilitated by sugammadex (2 mg/kg). Subsequently, the patient developed laryngospasm upon emergence from anesthesia, manifesting as stridor and wheezing, yet maintaining oxygen saturation levels above 95% with supplemental oxygen. Management involved positive pressure ventilation, intravenous lidocaine administration, and nebulized albuterol, resulting in the resolution of the laryngospasm within 10 minutes. Subsequent assessment through a postoperative chest X-ray revealed a minor right pneumothorax and pleural effusion, as shown in Figure 1, both of which were asymptomatic and deemed not in need of intervention. The patient's treatment regimen included initiation of an oral diet, cefazolin, and metronidazole.

**Figure 2:**
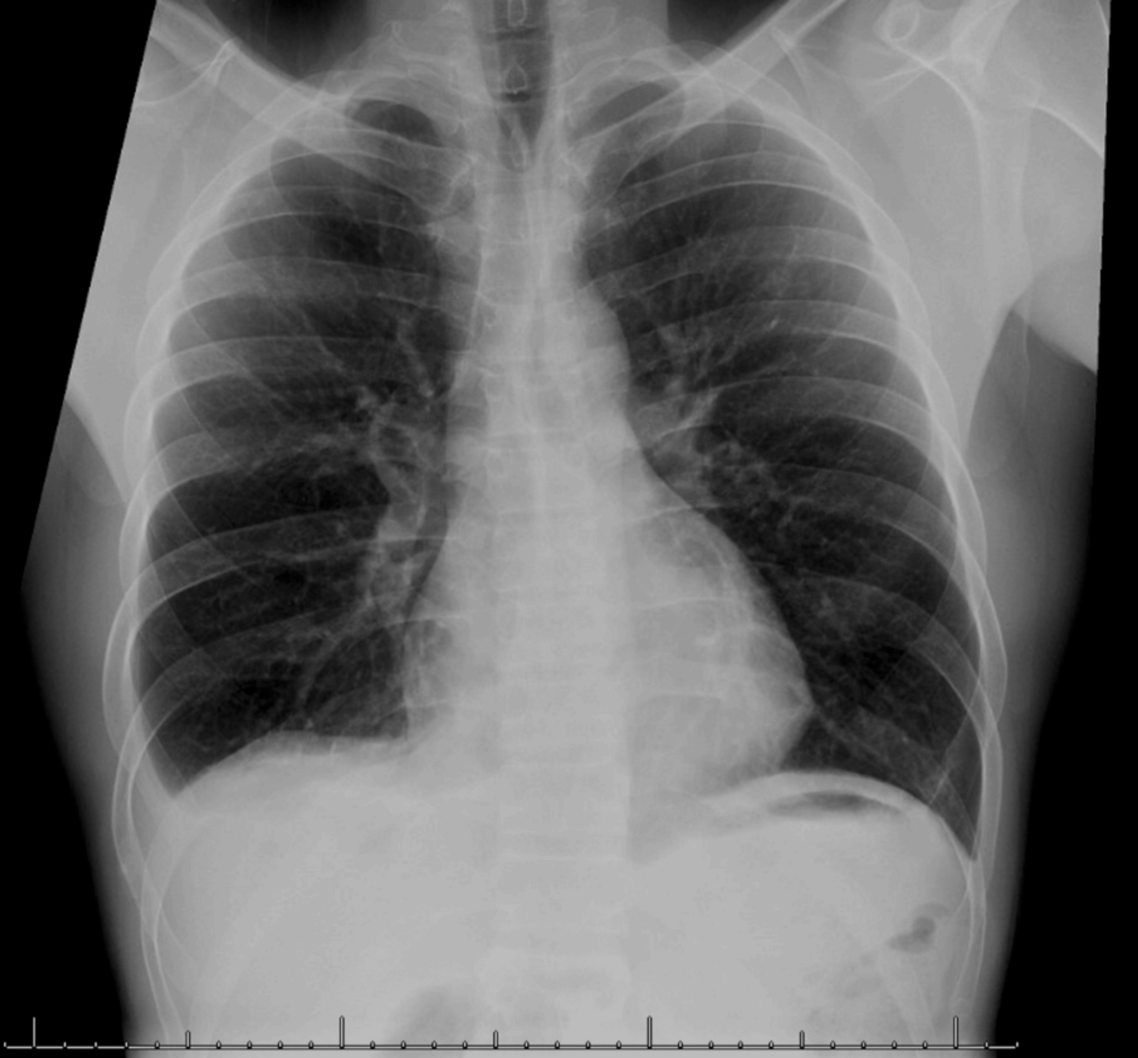
Chest X-ray showing the improvement of right pleural effusion and absence of pneumothorax.

On the third day following the surgical procedure, the patient exhibited sporadic episodes of fever reaching 102.5°F and tachycardia peaking at 110 beats per minute, notwithstanding the absence of any other noticeable symptoms. Despite lacking a known exposure history, a subsequent COVID-19 test yielded a positive result. Following this, the patient was transferred to the internal medicine department for further care. Elevated white blood cell counts at 13.4 combined with tachycardia and a fever curve prompted consultation with an infectious disease specialist. The differential included COVID-19, urosepsis, or a super-imposed bacterial pulmonary infection, and intravenous vancomycin, ceftriaxone, and remdesivir treatment regimen was initiated. Results from blood and urine cultures returned negative, while a repeat chest X-ray indicated no alterations in the pneumothorax and pleural effusion. The patient was isolated within a negative pressure environment, and a thorough contact tracing was carried out. Notably, the patient did not display any signs of respiratory distress or hypoxia, maintaining oxygen saturation levels above 95% while breathing ambient air, and remained devoid of other manifestations associated with COVID-19. The patient's fever and tachycardia resolved within two days, and he was switched to oral ciprofloxacin for a seven-day course to treat concomitant pyelonephritis. His pneumothorax and pleural effusion resolved spontaneously, followed by a clear chest X-ray on the seventh day post-surgery, as shown in Figure 2. He was discharged on the eighth day post-surgery, and follow-up by phone and telemedicine revealed no recurrence of fever, pain, or urinary symptoms. A COVID-19 test on day 21 post-surgery was negative. The stent was removed on day 28, and a renal ultrasound on day 42 showed no hydronephrosis or obstruction. The patient was asymptomatic and satisfied with his surgical outcome.

## Discussion

This case exemplifies an unusual clinical manifestation of COVID-19 infection complicated by laryngospasm in a 40-year-old male. Laryngospasm, characterized by the abrupt closure of the vocal cords resulting in airway blockage, is an infrequent complication of general anesthesia with a prevalence ranging from 0.4% to 5.8% [8]. Various factors, such as airway irritation, secretions, blood, foreign objects, extubation, or hypersensitivity reactions, can precipitate laryngospasm [9]. Recent findings indicate a potential association between laryngospasm and COVID-19 infection. A case study emphasized a severe and life-threatening paroxysmal laryngospasm in a COVID-19 patient, underscoring the necessity for healthcare providers to recognize this possible complication [10]. Additionally, inflammatory reactions in the laryngeal and tracheal tissues of individuals with COVID-19 have been documented, potentially predisposing them to laryngospasm. Histological examinations have revealed notable inflammatory infiltrates and vasculitis in the tracheal mucosa of COVID-19 patients, which might compromise the epithelial barrier and contribute to airway hyperactivity [11]. Management of laryngospasm typically involves positive pressure ventilation, intravenous lidocaine, nebulized albuterol, and intravenous propofol or muscle relaxants [12]. In this case, laryngospasm was likely induced by airway irritation from the endotracheal tube and may be related to the onset of COVID-19 infection. Nonetheless, the patient received successful treatment without encountering severe complications.

This case was also complicated by pneumothorax and pleural effusion, representing other infrequent complications of laparoscopic surgery, with reported incidences of 0.1-0.4% and 0.1-0.6%, respectively [13]. These complications may arise from direct trauma to the diaphragm or pleura, gas embolism, barotrauma, or elevated intrathoracic pressure as well as iatrogenic causes [14]. Strategies for prevention include meticulous trocar placement, avoidance of excessive insufflation pressure, and appropriate patient positioning [14]. The presence of COVID-19 infections can heighten these risks by causing substantial diffuse alveolar damage, thereby increasing the chances of alveolar rupture and subsequent air leakage into the pleural cavity, leading to pneumothorax [15]. Pleural effusions, though less frequent, may develop in COVID-19 patients due to augmented capillary permeability secondary to inflammatory responses [16].

COVID-19 infection introduces a new and evolving complication. It can be spread through respiratory droplets, aerosols, or contact with contaminated surfaces [17]. Preventive measures consist of screening, testing, isolation, and the utilization of personal protective equipment [18]. A study conducted by Tabourin et al. examined the nosocomial transmission of COVID-19 post-robotic surgical procedures, including laparoscopic surgeries; this revealed a significantly low incidence of postoperative COVID-19 infection when appropriate precautions were implemented [19]. This emphasizes that thorough preoperative screening and strict adherence to infection control protocols can reduce the risk of postoperative COVID-19 infection. In this case, despite a negative preoperative COVID-19 test and the absence of exposure history, the patient contracted COVID-19 post-surgery. The latent period of COVID-19 is generally estimated to be around five days, with some studies suggesting it could be as short as 2.5 days [20]. However, an early diagnosis was made, effective treatment was provided, and no respiratory or systemic complications ensued. This case emphasizes the significance of promptly diagnosing and managing COVID-19 infection in surgical patients, as well as upholding stringent infection control measures during the perioperative period.

## Conclusions

Laryngospasm might present as the initial clinical manifestation of COVID-19 infection postoperatively, following surgery, notwithstanding a negative preoperative COVID-19 test result. Laparoscopic surgeries are linked to severe adverse events, including laryngospasm, pneumothorax, and pleural effusion which could be exacerbated by COVID-19 infection. Timely identification and intervention play a crucial role in the management of these adverse events. This case study contributes valuable insights to the existing literature regarding the potential complications associated with laparoscopic surgery and the influence of COVID-19 on patients undergoing surgical interventions.
